# Emergence of carbapenem-resistant *Pseudomonas aeruginosa* ST179 producing both IMP-16 and KPC-2: a case study of introduction from Peru to Spain

**DOI:** 10.1128/spectrum.00614-24

**Published:** 2024-05-10

**Authors:** Joaquim Viñes, Carlos Lopera, Andrea Vergara, Ignasi Roca, Jordi Vila, Climent Casals-Pascual, José Antonio Martínez, Carolina García-Vidal, Alex Soriano, Cristina Pitart

**Affiliations:** 1 Servei de Microbiologia i Parasitologia-CDB, Hospital Clínic de Barcelona, Barcelona, Spain; 2 Institut de Salut Global (ISGlobal), Barcelona, Spain; 3 Servei Veterinari de Genètica Molecular (SVGM), Facultat de Veterinària, Universitat Autònoma de Barcelona, Bellaterra, Spain; 4 Departament de Malalties Infeccioses, Hospital Clínic de Barcelona, Barcelona, Spain; 5 Departament de Fonaments Clínics, Facultat de Medicina i Ciències de la Salut, Universitat de Barcelona, Barcelona, Spain; 6 CIBER Enfermedades Infecciosas (CIBERINFEC), Madrid, Spain; JMI Laboratories, North Liberty, Iowa, USA

**Keywords:** *Pseudomonas aeruginosa*, ST179, carbapenem-resistant, carbapenemase, IMP-16, KPC-2, KPC-35, nanopore, Illumina, leukemia

## Abstract

We describe four cases of a novel carbapenem-resistant *Pseudomonas aeruginosa* ST179 clone carrying the *bla*_KPC-2_ or *bla*_KPC-35_ gene together with *bla*_IMP-16_, imported from Peru to Spain and isolated from leukemia patients. All isolates were multidrug-resistant but remained susceptible to fosfomycin, cefiderocol, and colistin. Whole-genome sequencing revealed that *bla*_KPC-2_ and *bla*_KPC-35_ were located in an IncP6 plasmid, whereas *bla*_IMP-16_ was in a chromosomal type 1 integron. This study highlights the global threat of multidrug-resistant *P. aeruginosa* clones and underscores the importance of monitoring and early detection of emerging resistance mechanisms to guide appropriate treatment strategies. The importation and spread of such clones emphasize the urgent need to implement strict infection control measures to prevent the dissemination of carbapenem-resistant bacteria.

**IMPORTANCE:**

This is the first documented case of a *Pseudomonas aeruginosa* ST179 strain carrying the blaKPC-35 gene, and it represents the first report of a *P. aeruginosa* co-harboring blaIMP-16 and either blaKPC-2 or blaKPC-35, which wre imported from Peru to Spain, highlighting a threat due to the capacity of spreading carbapenem-resistance via plasmid conjugation.

## INTRODUCTION

*Pseudomonas aeruginosa* is a Gram-negative bacillus known for its adaptability and virulence and has emerged as a significant pathogen causing serious nosocomial infections, mainly affecting immunocompromised patients, especially those suffering from hematological malignancies such as acute leukemia (1).

Carbapenems, a class of broad-spectrum antibiotics, have long been the treatment of choice for *P. aeruginosa* infections. However, the emergence and spread of carbapenem resistance, especially through carbapenemase activity, have significantly limited treatment options. Carbapenemase-producing *P. aeruginosa* infections constitute a challenge for the treatment of these patients, leading to increased mortality (2). Genes encoding carbapenemases are usually found in mobile genetic elements, such as plasmids or transposons, and play a pivotal role in the spread of carbapenem resistance (3). The worldwide spread of high-risk clones represents a threat to global public health. In Central and South America, carbapenemase-producing *P. aeruginosa* represents 69% of all carbapenem-resistant *P. aeruginosa*, with KPC-2 and VIM-2 being the most prevalent carbapenemases found (4). Recent studies in Spain showed an increase in carbapenemase-producing *P. aeruginosa*, mainly due to the dissemination of high-risk clones, such as ST175 and ST244 producing VIM and IMP metallo-beta-lactamases, respectively (3, 5).

We describe the emergence of IMP-16, KPC-2, and KPC-35 carbapenemase-producing *P. aeruginosa*, isolated from three Peruvian patients who arrived from Peru and one Spanish patient with no recent travel history, who had shared a room with one of the Peruvian patients.

## MATERIALS AND METHODS

### Patients and isolates

From August 2022 to February 2023, five isolates of *P. aeruginosa* producing both KPC and IMP carbapenemases were recovered from either surveillance or clinical samples of four leukemic patients admitted to the hematology and ICU units at the Hospital Clínic of Barcelona.

### Strain identification, antimicrobial susceptibility testing, and detection of carbapenemases

Routine identification at the species level was performed using MALDI-TOF mass spectrometry (Bruker Daltonics GmbH & Co. KG, Bremen, Germany). Antimicrobial susceptibility testing was performed using automated methods (Phoenix M50, Becton–Dickinson, New York, USA), diffusion gradient strips for cefiderocol testing (Liofilchem, Roseto degli Abruzzi, Italy), and microdilution UMIC strips (Bruker Daltonics, Bremen, Germany) for colistin testing. Interpretative breakpoints were based on the European Committee on Antimicrobial Susceptibility Testing (EUCAST v13.0). The detection of KPC, OXA-48 like, IMP, VIM, and NDM carbapenemase production was performed using the lateral flow assay NG-Test CARBA 5 (NG-BIOTECH, France).

### DNA extraction and Illumina and Oxford Nanopore sequencing

DNA was extracted using the ZymoBIOMICS DNA miniprep Kit (Zymo Research, Irvine, USA), according to the manufacturer’s protocol. DNA quality and quantity were determined using NanoDrop 2000 spectrophotometer and Quantus Fluorometer and QuantiFluor dsDNA System (Promega, Madison, USA), respectively.

Illumina libraries were prepared with the Illumina DNA Prep kit (Illumina, San Diego, USA) and Nextera DNA CD Indexes (Illumina, San Diego, USA) using approximately 400 ng of input DNA. The libraries were normalized using a bead-based procedure and were pooled at equal volumes. The pooled library was denatured and sequenced using MiSeq reagent version 2 (Illumina, San Diego, USA) on an Illumina MiSeq platform with a 2 × 150 paired-end chemistry.

Oxford Nanopore Technologies (ONT) libraries were prepared using approximately 400 ng of DNA with the Rapid Barcoding Sequencing kit (SQK-RBK004; ONT, Oxford, UK). The MinION FLO-MIN106 v9.4.1 flow cell (ONT, Oxford, UK) and MinION Mk1C device (ONT, Oxford, UK) were used for sequencing for approximately 48 hours.

### Genome assembly and quality assessment

Live basecalling was selected, and reads with a quality score lower than 8 and <200 bp were discarded. Hybrid assembly was performed as follows. A first assembly was created using ONT reads with Unicycler v0.5.0 (6). Subsequently, an ONT-based polishing step was performed using Medaka v.1.7.2 (7). Illumina FASTQ files were mapped to the polished assemblies using BWA v0.7.12 (8). A consensus Illumina-based polishing step was performed with Polypolish v0.5.0 (9), from which the final ONT-Illumina polished assemblies were obtained, except for 23–169, since no Illumina data were generated for this sample.

The completeness of the final assemblies was assessed using a double approximation of CheckM v1.2.2 (10) and BUSCO v5.3.2 (11). For a review of the assembly performance, see Supplementary Information.

### Genome analysis

Multilocus sequence typing (MLST) was performed using PubMLST (12). The presence of antibiotic resistance genes (ARGs), plasmid replicons, and virulence factors was assessed using ABRicate v1.0.1 (13) alongside CARD (14) and NCBI databases for ARGs; PlasmidFinder (15) database for plasmid replicons; and the VFDB database for carriage of virulence factors. The mutational resistome was analyzed by isolating the genes of interest [described by López-Causapé *et al*. (16)] and comparing them with he PAO1 reference using BioEdit (17).

Bandage (18) v0.8.1 was further used to map the *P. aeruginosa* reference genome PAO1 (accession number NC_002516.2) and the p10265 plasmid (a plasmid bearing the *bla*_KPC-2_ gene, accession number KU578314.1) against all contigs to differentiate between chromosomal and plasmid contigs bearing the *bla*_KPC-2_ gene.

Plasmid sequences were further studied using oriTFinder (19) to identify the origin of transfer (oriT), relaxase genes, and carriage of type 4 secretion systems (T4SS). Prokka v1.14.6 (20), GeneMarkS-2 v1.25 (21), BLAST (22), and ISfinder (23) were used for the genetic annotation and identification of putative mobile elements. SnapGene v5.2.4 (from Insightful Science; available at https://www.snapgene.com/) was used to generate genetic diagrams.

The average nucleotide identity (ANI) of the chromosomes was calculated using FastANI (24) v1.33. Single-nucleotide polymorphisms (SNPs) for inferring phylogeny were called using CSI Phylogeny (25) v1.4. The Newick file obtained was visualized using FigTree (http://tree.bio.ed.ac.uk/software/figtree/) v1.4.4. For FastANI and CSI Phylogeny analysis, 20 other *P. aeruginosa* chromosomes from the NCBI were included: nine ST179 (including one from Peru, one from Canada, three from Australia, and four from the United Kingdom) and other STs around the world (one from Costa Rica, one from Canada, one from Germany, three from the USA, and five from Peru): 17387, 17469, A12, AG1, AMar-01, AUSMDU00006763, AUSMDU00007189, AUSMDU00007875, B34, C108, C115, C4.2, Cli-EG-01, Cli-Tap-01, NSC1791, PA96, PAC1, PAO1, SK010, and UCBPP-PA14. Chromosomal integrons were described using IntegronFinder 2.0 (26).

## RESULTS

### Medical history

Patient 1 was an 81-year-old male born in Spain and residing in Barcelona. He was diagnosed with acute myeloid leukemia (AML) in June 2022 and started treatment at our center in July 2022.

Patient 2 was a 23-year-old male born in Peru. He was diagnosed with early T acute lymphoblastic leukemia (ALL) in December 2017 in Peru. On the 6th of July 2022, immediately after arriving in Spain from Peru, he was admitted to the emergency unit of our center because of fever and cutaneous lesions compatible with ecthyma gangrenosum.

Patient 3 was a 23-year-old male born in Peru diagnosed with ALL in August 2017 and underwent induction treatment between August 2017 and March 2020. In July 2022, the patient’s symptoms worsened, and he did not respond to treatment with cytarabine and mitoxantrone. He traveled to Spain to obtain a second medical opinion and was admitted to the emergency unit of our center on the 17th of August because of fever.

Patient 4 was a 65-year-old woman who was native to Cuzco, Peru. She had been residing in Barcelona for over a month before being admitted to the emergency unit of our center. In June 2022, she was diagnosed with ALL and monitored in Peru. She was currently receiving bimonthly supportive treatment with 6-mercaptopurine. Upon admission to our center on the 2nd of February 2023, the patient suffered from a symptomatic central nervous system affection. On 20th February, she was transferred to the ICU because of respiratory-origin septic shock.

Patient 1 shared a two-bed hospital room with patient 2 when a carbapenem-resistant *P. aeruginosa* isolate producing both IMP metallo-beta-lactamases and a KPC-type carbapenemase was recovered first from a urine sample and a surveillance sample in patient 1 and also from a wound sample from patient 2. Subsequent isolates with a similar phenotype and carrying carbapenemase-encoding genes were also recovered from patients 3 and 4 (see Table 1).

**TABLE 1:** Isolates and patients included in this study

Patient ID	Patient nationality	Ward	Admission date	Discharge date	Isolate	Sample collection date	Sample
1	Spanish	Hematology	26/07/2022	26/08/2022	NA^*a*^	13/08/2022	Urine
					22–690	16/08/2022	Rectal swab
2	Peruvian	Hematology	06/07/2022	14/09/2022	22–722	24/08/2022	Wound
					22–841	07/10/2022	Wound
3	Peruvian	Hematology	17/08/2022	01/12/2022	22–969	15/11/2022	Urine
4	Peruvian	ICU	2/02/2023	4/05/2023	23–169	21/02/2023	Rectal swab

^*a*^ NA: isolate not available for the study.

### Antibiotic susceptibility profile

Susceptibility testing showed that all isolates were resistant to aminoglycosides, cephalosporins, carbapenems, and novel beta-lactam/beta-lactamase inhibitor combinations, such as ceftazidime/avibactam and ceftolozane/tazobactam, whereas they were only susceptible to cefiderocol, fosfomycin, and colistin (Table 2).

**TABLE 2:** Antimicrobial susceptibility profile of *P. aeruginosa* isolates from this study

	MIC (µg/mL) (category^*a*^)				
Isolates	22–690	22–722	22–841	22–969	23–169
Ceftazidime	>16 (R)	>16 (R)	>16 (R)	>16 (R)	>16 (R)
Cefepime	>16 (R)	>16 (R)	>16 (R)	>16 (R)	>16 (R)
Piperacillin–tazobactam	>32/4 (R)	>32/4 (R)	>32/4 (R)	>32/4 (R)	>32/4 (R)
Aztreonam	>32 (R)	>32 (R)	>32 (R)	>32 (R)	>32 (R)
Cefiderocol	0.19	0.19	0.75	0.75	0.5
Ceftazidime/avibactam	>16 (R)	>16 (R)	>16 (R)	>16 (R)	>16 (R)
Ceftolozane/tazobactam	>16 (R)	>16 (R)	>16 (R)	>16 (R)	>16 (R)
Imipenem	>8 (R)	>8 (R)	>8 (R)	>8 (R)	>8 (R)
Meropenem	>16 (R)	>16 (R)	>16 (R)	>16 (R)	>16 (R)
Gentamicin	>8 (R)	>8 (R)	>8 (R)	>8 (R)	>8 (R)
Tobramycin	>8 (R)	>8 (R)	>8 (R)	>8 (R)	>8 (R)
Amikacin	>32 (R)	>32 (R)	>32 (R)	>32 (R)	>32 (R)
Ciprofloxacin	>2 (R)	>2 (R)	>2 (R)	>2 (R)	>2 (R)
Fosfomycin	8 (S)	8 (S)	8 (S)	8 (S)	8 (S)
Colistin	1 (S)	1 (S)	1 (S)	1 (S)	1 (S)

^*a*^ Categories were assigned according to EUCAST 2023 guidelines: R, resistant; I, susceptible increased exposure; S, susceptible standard dosing regimen.

### Genomic analysis

**Fig 1:**
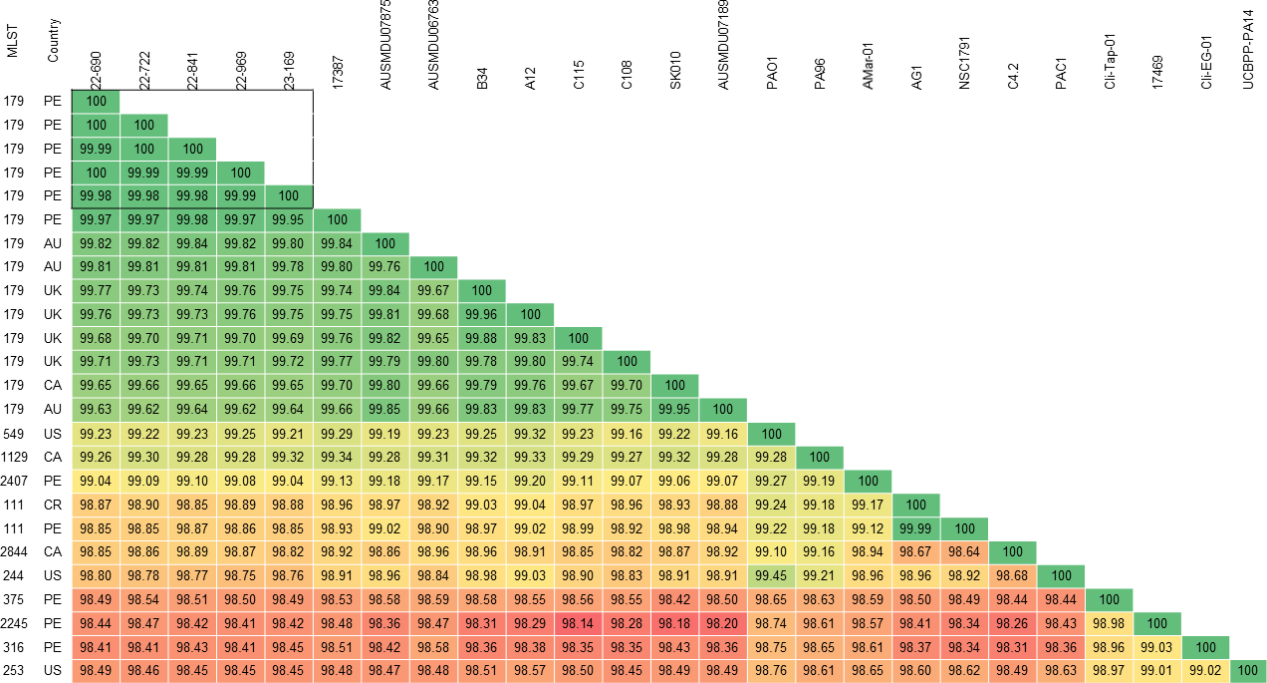
Average nucleotide identity (ANI) of *P. aeruginosa* isolates and reference genomes. AU, Australia; CA, Canada; CR, Costa Rica, PE, Peru; UK, United Kingdom; US, United States.

All isolates belonged to sequence type ST179. ANI demonstrated that nucleotide identity between the five *P. aeruginosa* isolates ranged from 99.98% to 100% (Fig. 1). Interestingly, a IMP-16 *P. aeruginosa* ST179 isolate (17387) collected in Lima, Peru, in 2021 and submitted at the NCBI in 2022 (27), presented identity values with the isolates from this study that ranged from 99.95% to 99.98%, while the ANI values of additional ST179 isolates retrieved from NCBI and recovered in Australia, United Kingdom, and Canada ranged from 99.62% to 99.82%. Isolates belonging to sequence types other than ST179 had ANI values ranging from 98.41% to 99.32%.

**Fig 2:**
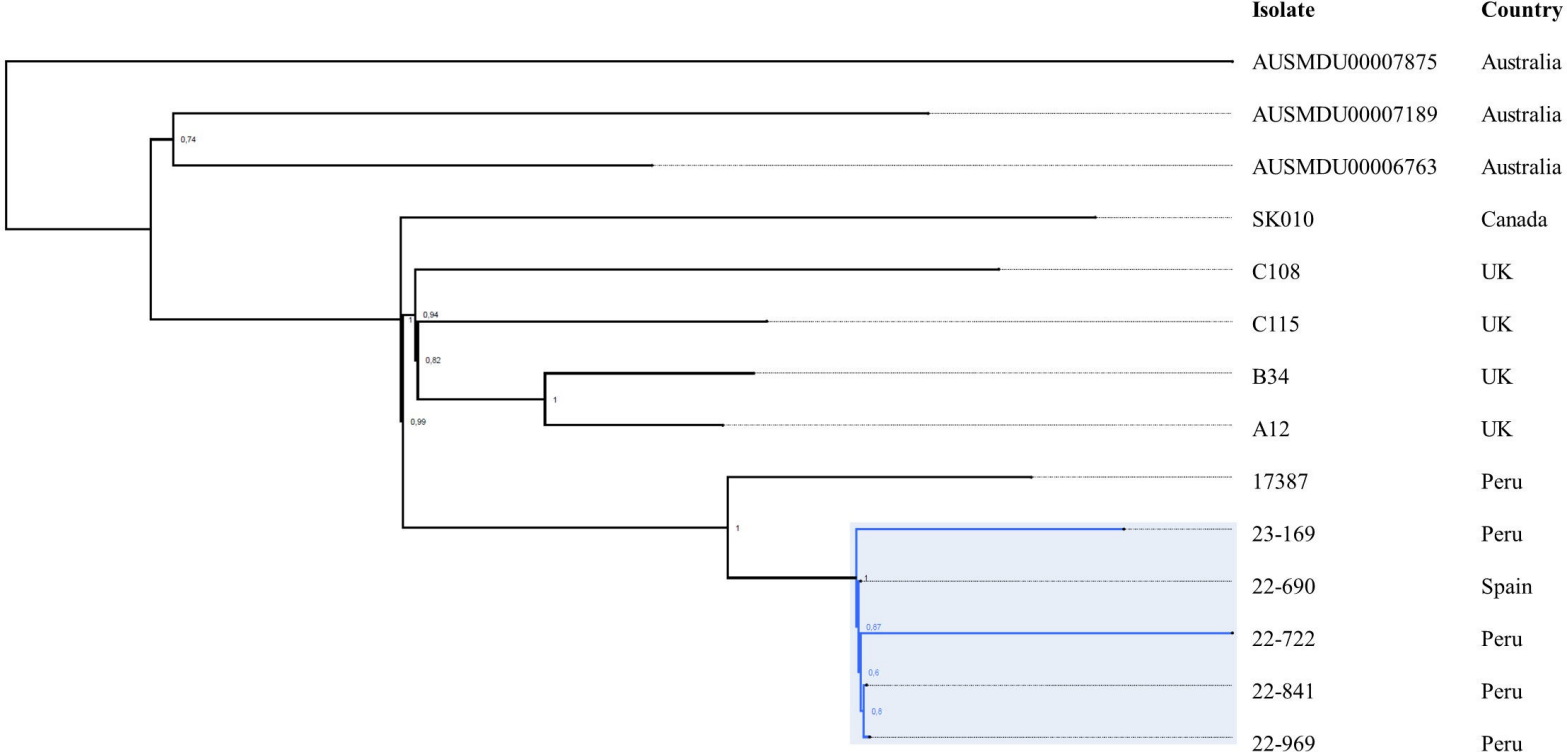
SNP-based phylogenetic tree of ST179 *P. aeruginosa* isolates. The group of isolates in this study is highlighted in blue. Image visualized with FigTree.

Phylogenetic analysis based on single-nucleotide polymorphisms (SNPs) of the five *P. aeruginosa* isolates recovered in the study and the genomes retrieved from the NCBI showed a cluster of all ST179 isolates, as expected, with isolates recovered in this study being closely related and clustered together with isolate 17387, recovered from a Peruvian patient in 2021, in good agreement with results from the ANI analysis (Fig. 2).

**Fig 3:**
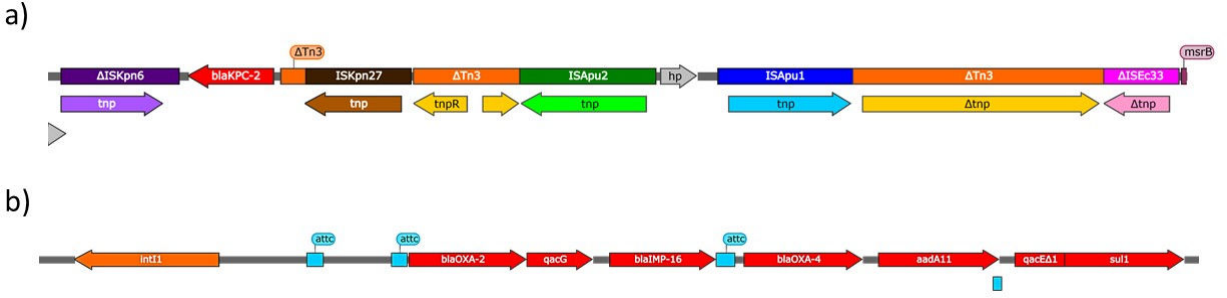
Genetic context for (a) *bla*_KPC-2_ and *bla*_KPC-35_ genes and (b) the *bla*_IMP-16_ gene. Image visualized with SnapGene.

*In silico* analysis described 15 antibiotic resistance genes (ARGs) (Table S1), highlighting the presence of genes encoding metallo-beta-lactamase IMP-16 and class A carbapenemases KPC-2 and KPC-35. *bla*_KPC_ genes were harbored in plasmids belonging to the IncP6 incompatibility group (see below). The *bla*_IMP-16_ gene was located within a type 1 integron structure and was flanked by additional ARGs, such as *bla*_OXA-2_, *bla*_OXA-4_, and *aadA11* (Fig. 3). The mutational resistome of the strains was studied (Table S2) as described by López-Causapé *et al*. (16).

### IncP6 plasmid harboring the *bla*_KPC_ gene

The IncP6 plasmid of approximately 34,699 bp (ranging from 34,641 bp to 34,726 bp) and 57.7% GC carried the *bla*_KPC-2_ allele in four isolates (22–690, 22–722, 22–969, and 23–169), but the *bla*_KPC-35_ allele in isolate 22–841. All IncP6 plasmids also carried an origin of transference, the *repA* gene coding for a replicase, and additional genes such as the partition system *parABC* and the relaxosome and mobilization genes *mobABCDE*, but lacked the genes encoding a type IV secretion system that is essential for conjugation, thus suggesting that it was not self-transferable. Genes encoding for a type IV secretion system were present in all isolates within another contig.

The *bla*_KPC_ genes were located in the same plasmid and genetic context among the five isolates, which was not the canonical *Tn4401* element typically associated with *bla*_KPC_ but consisted of a composite transposon of approximately 17 kbp containing an IS*Kpn27-bla*_KPC-2_-ΔIS*Kpn6* core structure as well as *Tn3-*associated structures, as previously described in *Klebsiella pneumoniae* isolates recovered from the same hospital in 2018 (28) (Fig. 3).

## DISCUSSION

We describe the potential emergence and transmission of IMP-16, KPC-2, or KPC-35 carbapenem-resistant *P. aeruginosa* ST179 clones imported from Peru from advanced stage leukemia-colonized patients admitted to a Spanish hospital in Barcelona. We included five isolates from four patients with different types of leukemia. Interestingly, three of the four patients were from Peru and had just recently traveled to Spain, and two of them shared the same hematological ward together with the only patient from Spain, with whom one of them had also shared a two-bed hospital room when the first carbapenem-resistant *P. aeruginosa* isolate was recovered.

Halat *et al*. reviewed the current status and global epidemiology of carbapenemases in *P. aeruginosa* in 2022 (29), which included several carbapenemase genes such as *bla*_KPC_*, bla*_GES_*, bla*_VIM_*, bla*_IMP_*, bla*_NDM_, and *bla*_OXA_ variants, and stated that in countries such as Brazil, Peru, and Costa Rica, the rates of carbapenem-resistant *P. aeruginosa* were higher than 50%. In Peru, carbapenem-resistant *P. aeruginosa* varies geographically, being more reported in the Lima region. According to Angles-Yanqui *et al*., the predominant carbapenemase gene is *bla*_IMP_, while *bla*_NDM_ and *bla*_KPC_ are more frequently found among *Enterobacterales* (30). In Spain, the prevalence of KPC and IMP carbapenemases is not prominent. Nevertheless, various IMP carbapenemases have been reported in the region, including IMP-11 in *Acinetobacter baumannii* and IMP-28 in *Enterobacter kobei* in A Coruña (31), IMP-13 in *Enterobacter cloacae* (32), IMP-16 and IMP-23 in *P. aeruginosa*, and IMP-22 in *Enterobacter* in Andalusia (33). Moreover, IMP-8, IMP-8 +OXA-48, and IMP-22 +KPC-3 *K. pneumoniae* strains were identified at the Instituto de Salud Carlos III (34). In 2021, Salvador-Luján *et al*. reported for the first time the presence of the *bla*_KPC_ gene in a *P. aeruginosa* strain isolated from the urine of a 56-year-old male in Peru (35). Tickler *et al.* (27) also investigated the epidemiology of Peruvian carbapenem-resistant *P. aeruginosa* isolates and found that ST179 was the third most frequently encountered sequence type (*n* = 12, 8.5%) after ST111 and ST357 (44% and 38.3%, respectively). Of the 12 ST179 isolates recovered in 2018 (*n* = 8) and 2021 (*n* = 4), only two of them recovered in 2018 harbored the *bla*_IMP-16_ gene, and no carriage of *bla*_KPC_ was reported. Moreover, ST179 presented the virulence profile *exoS*+/*exoT*+/*exoY*+ (T3SS), which was also shared with all the isolates described in our study. All isolates presented the same virulence factor profile (Table S3), which included the coding regions for a type 3 and a type 6 secretion system (T3SS and T6SS, respectively), two metallophores (pyoverdine and pyochelin), pili, flagella, and exotoxin A, which are all related with the pathogenesis of *P. aeruginosa*. T3SS and T6SS are involved in the translocation of secreted toxins that enhance disease severity, such as ExoS/T/Y secreted effector toxins, coded in the isolates from this study. For example, ExoS causes apoptotic cell death (36–38). Pyoverdine and pyochelin scavenge metals such as iron from the host (39).

The *P. aeruginosa* clone in this study carried the *bla*_IMP-16_ metallo-beta-lactamase gene and *bla*_KPC-2_ or *bla*_KPC-35_ carbapenemase genes. KPC-2 is one of the predominant KPC carbapenemases that has spread worldwide, including Latin America and Spain (40). The *bla*_KPC-35_ gene is an allelic variant of *bla*_KPC-2_ that differs in only one nucleotide mutation that translates into an L169P amino acid substitution located in the omega loop of the KPC-2 protein, conferring resistance to ceftazidime/avibactam, and is usually associated with ceftazidime/avibactam treatment in patients infected with carbapenem-resistant *K. pneumoniae* isolates producing KPC-2 variants (41–44).

In this study, a *P. aeruginosa* isolate producing KPC-35 (22-841) was recovered from a patient previously carrying a clonally related isolate that harbored the *bla*_KPC-2_ allele and received antimicrobial treatment with ceftazidime/avibactam, thus most likely promoting the emergence of the mutation in the *bla*_KPC-2_ sequence that gave place to *bla*_KPC-35_. However, as this *P. aeruginosa* isolate also carried metallo-beta-lactamase *bla_I_*_MP-16_, we could not detect any changes in the MIC values against ceftazidime/avibactam. To our knowledge, this is the first report of a *P. aeruginosa* isolate carrying the *bla*_KPC-35_ variant, as well as the first time that a *P. aeruginosa* strain is accounted for simultaneously producing KPC-2 or KPC-35 together with IMP-16.

Interestingly, the genetic region containing the *bla*_KPC_ gene includes different insertion sequences located within a *Tn3* structure, as depicted in Fig. 3, including the IS*Kpn27*, IS*Apu2*, and IS*Apu1* insertion sequences, all flanked by incomplete ΔIS*Kpn6* and ΔIS*Ec33* insertion sequences. The KPC plasmid described in this study is very similar to the IncP6 plasmid described by Dai *et al*. (45), p10265-KPC (~38.9 kbp, accession number KU578314.1 differing only by two missing regions: (i) p10265-KPC presents a *Tn5563* transposon with a *tnpR–orf2–pilT–tnpA–merP–merT–merR* structure that in all isolates from this study lacks a fragment of the *pilT* gene (only 43.34% of coverage), the full *tnpA* gene, and the full *merP* gene; and (ii) the plasmid in our study also lacks the *Δbla*_TEM-1_ gene that is located next to *bla*_KPC_ in p10265-KPC.

The *P. aeruginosa* isolates included in this study had the *bla*_IMP-16_ gene located within a type 1 integron in the chromosome (Fig. 3). IMP-16 carbapenemase was first described in 2004 by Mendes *et al*. (46) in a *P. aeruginosa* isolate obtained from a 60-year-old man in Brazil and, to our knowledge, it has not been previously reported in Spain until now, most likely being introduced by Peruvian patients admitted to our hospital. The genetic content of the integron carrying *bla*_IMP-16_ differs from that described by Mendes *et al*.; the integron contained three aminoglycoside resistance genes (*aac(6’)−30*, *aac(6’)-Ib’*, and *aadA1*), whereas the one in our study only contained the *aadA11* gene.

In conclusion, to the best of our knowledge, this is the first documented case of a *Pseudomonas aeruginosa* ST179 strain carrying the *bla*_KPC-35_ gene, and it represents the first report of a *P. aeruginosa* strain co-harboring *bla*_IMP-16_ and either *bla*_KPC-2_ or *bla*_KPC-35_. The importation and spread of this clone from Peru to Spain emphasizes the critical need for monitoring carbapenemase-resistant bacteria as it poses significant challenges by limiting the available antibiotic options for treating infections. Furthermore, the potential emergence of new resistance mechanisms against new agents used to combat carbapenem-resistant microorganisms highlights the urgent need to promptly identify and describe such resistance to guide appropriate treatment, rather than relying on empirical approaches. Data from this study suggest that KPC/IMP-16-producing *P. aeruginosa* ST179 is either widespread in Peru or that all three patients from Peru might have been admitted at the same particular hospital where this strain is prevalent. Unfortunately, all efforts to gather additional information regarding previous admissions of such patients in hospital at their country of origin were unsuccessful, which we acknowledge as one of the main limitations of the study.

## Data Availability

Illumina and Nanopore FastQ read files were submitted to the Sequence Read Archive (SRA) from the National Center for Biotechnology Information (NCBI) under BioProject PRJNA984723. The GenBank accession numbers for the genome assemblies are as follows: 22–690 JAUAWL000000000.1; 22–722 JAUAWI000000000.1; 22–841 JAUAWJ000000000.1; 22–969 JAUAWK000000000.1; and 23–169 JAUAWL000000000.1.

## SUPPLEMENTAL MATERIAL

Supplemental material spectrum.00614-24-s0001.docx Information regarding the isolates within the study.

Supplemental tables spectrum.00614-24-s0002.xlsx Tables S1-S3.

ASM does not own the copyrights to Supplemental Material that may be linked to, or accessed through, an article. The authors have granted ASM a non-exclusive, world-wide license to publish the Supplemental Material files. Please contact the corresponding author directly for reuse.
