# Supplementary material for: Emergence of carbapenem-resistant Pseudomonas aeruginosa ST179 producing both IMP-16 and KPC-2: a case study of introduction from Peru to Spain
Source: Microbiol Spectr. 2024 May 10;12(6):e00614-24. doi: 10.1128/spectrum.00614-24 (PMC11237478; doi:10.1128/spectrum.00614-24)
Supplement: Supplemental material — Information regarding the isolates within the study. [file spectrum.00614-24-s0001.docx]

**Supplementary Information**

**SUPPLEMENTARY RESULTS**

**Assembly analysis**

**Supplementary Information Table 1** shows the assembly performance on chromosomes. Size ranged from 7,459,831 bp to 7,508,998 bp (average 7,485,513 bp) and CDS number ranged from 6,889 to 7,032 (average 6,946). All chromosomes presented a GC content of proximately 65.5% and CheckM and BUSCO completeness of 100%. All isolates presented two contigs belonging to two different plasmids, which will be further discussed at the “Plasmid content” section.

**Supplementary Information Table 1. Assembly performance on chromosomes**. Data from columns 2 to 7 was retrieved from Prokka. bp, base pairs; Ctgs, contigs; CheckM Compl., completeness.

|  |  |  |  |  |  |  | **CheckM** | **BUSCO** |  |  |
| --- | --- | --- | --- | --- | --- | --- | --- | --- | --- | --- |
| **Isolate** | **Ctgs** | **Size (bp)** | **CDS** | **rRNA** | **tRNA** | **tmRNA** | **Compl. (%)** | **Complete** | **GC%** | **Plasmids** |
| 22-690 | 2 | 7,483,488 | 6,929 | 12 | 75 | 1 | 99.68 | 100 | 65.57 | 2 |
| 22-722 | 11 | 7,508,998 | 6,950 | 13 | 77 | 1 | 99.68 | 100 | 65.48 | 2 |
| 22-841 | 7 | 7,497,358 | 6,931 | 12 | 75 | 1 | 99.68 | 100 | 65.60 | 2 |
| 22-969 | 3 | 7,459,831 | 6,889 | 12 | 75 | 1 | 99.68 | 100 | 65.52 | 2 |
| 23-169 | 4 | 7,477,889 | 7,032 | 12 | 75 | 1 | 99.68 | 100 | 65.38 | 2 |

**Antibiotic resistance and virulence genes**

*In silico* analysis described 15 antibiotic resistance genes (ARGs): five aminoglycoside resistance genes (*aac(6')-Il*, *aadA11*, *ant(22’’)-Ia*, *aph(3’)-IIb*, and *aph(3’)-Via*); seven genes coding for beta-lactamases (*bla_OXA-2_*, *bla_OXA-4_*, *bla_OXA-396_*, and *bla_PDC-8_* as well as *bla_IMP-16_*, *bla_KPC-2_*, and *bla_KPC-35_* coding for carbapenemases); the chloramphenicol resistance gene *catB7*; the *crpP* gene involved in ciprofloxacin resistance; and *sul1* for resistance to sulfonamides. All ARGs were located within the bacterial chromosome, except for the *bla_KPC_* genes.

All isolates also presented the same profile of virulence genes (**Supplementary table 3**), that included 226 genes encoding for type III and type VI secretion systems, the siderophores pyochelin and pyoverdine, flagella and fimbria (type IV pili), as well as exotoxin A, among others.
